# Protective Efficacy of Inhaled BCG Vaccination Against Ultra-Low Dose Aerosol *M. tuberculosis* Challenge in Rhesus Macaques

**DOI:** 10.3390/pharmaceutics12050394

**Published:** 2020-04-25

**Authors:** Andrew D. White, Charlotte Sarfas, Laura S. Sibley, Jennie Gullick, Simon Clark, Emma Rayner, Fergus Gleeson, Martí Català, Isabel Nogueira, Pere-Joan Cardona, Cristina Vilaplana, Mike J. Dennis, Ann Williams, Sally A. Sharpe

**Affiliations:** 1 Public Health England, National Infection Service, Porton Down, Salisbury SP4 0JG, UK; charlotte.sarfas@phe.gov.uk (C.S.); laura.sibley@phe.gov.uk (L.S.S.); J.Gullick@soton.ac.uk (J.G.); simon.clark@phe.gov.uk (S.C.); emma.rayner@phe.gov.uk (E.R.); mike.dennis@phe.gov.uk (M.J.D.); ann.rawkins@phe.gov.uk (A.W.); sally.sharpe@phe.gov.uk (S.A.S.); 2 The Churchill Hospital, Headington, Oxford OX3 7LE, UK; fgleeson@icloud.com; 3 Comparative Medicine and Bioimage Centre of Catalonia (CMCiB), Fundació Institut d’Investigació en Ciències de la Salut Germans Trias i Pujol, Badalona, 08916 Catalonia, Spain; marticatalasabate@gmail.com; 4 Servei de Radiodiagnòstic, Hospital Universitari Germans Trias i Pujol, Badalona, 08916 Catalonia, Spain; inogueiram@gmail.com; 5 Unitat de Tuberculosi Experimental, Fundació Institut d’Investigació en Ciències de la Salut Germans Trias i Pujol, Universitat Autònoma de Barcelona, CIBERES, 28029 Madrid, Spain; pj.cardona@gmail.com (P.-J.C.); cvilaplana@gmail.com (C.V.); 6 Centro de Investigación Biomédica en Red de Enfermedades Respiratorias (CIBERES). Av. Monforte de Lemos, 3-5. Pabellón 11. Planta 0. 28029 Madrid, Spain

**Keywords:** tuberculosis, BCG, vaccine, non-human primate, macaque, aerosol vaccination, immunogenicity, T-cell memory

## Abstract

Ten million cases of tuberculosis (TB) were reported in 2018 with a further 1.5 million deaths attributed to the disease. Improved vaccination strategies are urgently required to tackle the ongoing global TB epidemic. In the absence of a validated correlate of protection, highly characterised pre-clinical models are required to assess the protective efficacy of new vaccination strategies. In this study, we demonstrate the application of a rhesus macaque ultra-low dose (ULD) aerosol *M. tuberculosis* challenge model for the evaluation of TB vaccination strategies by directly comparing the immunogenicity and efficacy of intradermal (ID) and aerosol BCG vaccination delivered using a portable vibrating mesh nebulizer (VMN). Aerosol- and ID-delivered Bacille Calmette-Guérin (BCG) induced comparable frequencies of IFN-γ spot forming units (SFU) measured in peripheral blood mononuclear cells (PBMCs) by ELISpot, although the induction of IFN-γ SFU was significantly delayed following aerosol immunisation. This delayed response was also apparent in an array of secreted pro-inflammatory and chemokine markers, as well as in the frequency of antigen-specific cytokine producing CD4 and CD8 T-cells measured by multi-parameter flow cytometry. Interrogation of antigen-specific memory T-cell phenotypes revealed that vaccination-induced CD4 and CD8 T-cell populations primarily occupied the central memory (TCM) and transitional effector memory (TransEM) phenotype, and that the frequency of CD8 TCM and TransEM populations was significantly higher in aerosol BCG-vaccinated animals in the week prior to *M. tuberculosis* infection. The total and lung pathology measured following *M. tuberculosis* challenge was significantly lower in vaccinated animals relative to the unvaccinated control group and pathology measured in extra-pulmonary tissues was significantly reduced in aerosol BCG-vaccinated animals, relative to the ID-immunised group. Similarly, significantly fewer viable *M. tuberculosis* CFU were recovered from the extra-pulmonary tissues of aerosol BCG-vaccinated macaques relative to unvaccinated animals. In this study, a rhesus macaque ULD *M. tuberculosis* aerosol challenge model was applied as a refined and sensitive system for the evaluation of TB vaccine efficacy and to confirm that aerosol BCG vaccination delivered by portable VMN can confer a significant level of protection that is equivalent, and by some measures superior, to intradermal BCG vaccination.

## 1. Introduction

An estimated 10 million incident cases of *Mycobacterium tuberculosis* infection were reported in 2018, and 1.5 million deaths were attributed to tuberculosis (TB) disease [1]. Ten percent of new TB cases occurred in people with HIV and drug-resistant strains of *M. tuberculosis* were responsible for approximately 500,000 infections during this time; hence, there remains an urgent need for improved vaccination strategies to tackle the ongoing global TB epidemic. In the absence of a validated correlate of protection, highly characterised pre-clinical models are required to assess the protective efficacy of new vaccination strategies. The disease that develops in non-human primates (NHPs) following *M. tuberculosis* exposure closely resembles the tuberculous disease observed in humans, and the similarities between NHP and human anatomy, physiology, and immune response make primate models the most relevant for the assessment of TB vaccines [2,3,4,5]. However, the choice of *M. tuberculosis* challenge strain and dose can influence the level of disease that develops, posing the risk that promising new vaccines may be disregarded due to a failure to protect against a challenge inoculum that is unrepresentative of natural infection [4,5]. We have recently established an ultra-low dose (ULD) aerosol challenge system using the *M. tuberculosis* Erdman strain that produces consistent and measurable levels of disease in both rhesus and cynomolgus macaques [6]. In this study, we demonstrate the application of the ULD *M. tuberculosis* challenge model in rhesus macaques for the evaluation of vaccine efficacy and use the measurement of bacterial burden, qualitative gross pathology, histopathology and advanced in vivo imaging as readouts of vaccination mediated modulation of disease burden.

The attenuated strain of *Mycobacterium bovis*, Bacille Calmette-Guérin (BCG), was first used in the 1920s and remains the only licensed prophylactic TB vaccine [1]. Delivered as an intradermal (ID) injection, BCG is known to reduce the occurrence of severe childhood forms of TB [7] but provides limited and geographically variable protection against the infectious pulmonary manifestation of TB disease in adults [8,9]. In addition to a portfolio of novel TB vaccine candidates that are progressing through clinical trials [1], alternative BCG delivery strategies such as revaccination [10,11] and vaccination via alternative delivery routes are currently under investigation [12]. The protection imparted by BCG vaccination against experimental *M. tuberculosis* infection has been shown to be enhanced when the vaccine is delivered by aerosol to mice [13], guinea pigs [14,15], and rhesus macaques [16]. Historically, the size and complexity of the apparatus required to generate and deliver viable bacterial aerosols in a controlled and consistent manner have hindered the deployment of aerosol BCG vaccination strategies. However, advances in nebuliser technology, and, specifically, the advent of portable vibrating mesh nebulisers (VMN) [17,18], has made the prospect of mass aerosol BCG vaccination campaigns a more practical proposition. We have previously demonstrated the safety of aerosolised BCG vaccination delivered using the Omron U22 VMN and the capacity of this approach to generate robust mucosal and systemic immune responses in a dose-dependent manner [19]. In this study, we directly compare the immunogenicity and efficacy of BCG vaccination delivered by aerosol or ID injection in the rhesus macaque ULD *M. tuberculosis* challenge model and demonstrate the value of the model as a platform for the assessment of novel TB vaccine candidates and strategies.

## 2. Materials and Methods

### 2.1. Experimental Animals

Nineteen rhesus macaques (*Macaca mulatta*) of Indian origin aged between 3.7 and 3.9 years of age were sourced from an established, closed UK breeding colony. Absence of previous exposure to mycobacterial antigens was confirmed by tuberculin skin test as part of colony management procedures and by screening for IFN-γ ELISpot (MabTech, Nacka. Sweden) responses to tuberculin-PPD (purified protein derivative) (SSI, Copenhagen, Denmark), and pooled 15-mer peptides of ESAT6 and CFP10 (Peptide Protein Research LTD, Fareham, UK).

Compatible social groups were housed in accordance with the Home Office (UK) Code of Practice for the Housing and Care of Animals Used in Scientific Procedures (1989), and the National Committee for Refinement, Reduction and Replacement (NC3Rs) Guidelines on Primate Accommodation, Care and Use, August 2006 (NC3Rs, 2006). Cages were approximately 2.5 m high by 4 m long by 2 m deep, constructed with high-level observation balconies and with a floor of deep litter to allow foraging. Additional environmental enrichment was afforded by the provision of toys, swings, feeding puzzles, and DVDs for visual stimulation. In addition to standard old-world primate pellets, diet was further supplemented with a selection of fresh vegetables and fruit. For each procedure, sedation was applied by intramuscular injection with ketamine hydrochloride (10 mg/kg) (Ketaset, Fort Dodge Animal Health Ltd, Southampton, UK). None of the animals had been used previously for experimental procedures, and each socially compatible group was randomly assigned to a particular study treatment. The study design and all procedures were approved by the Public Health England, Porton Down Animal Welfare and Ethical Review Body, and authorised under an appropriate UK Home Office project license.

**Figure 1:**
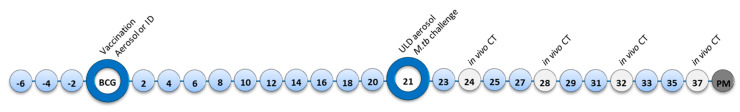
Study timeline relative to aerosol or ID BCG vaccination. Rhesus macaques received BCG vaccination delivered by aerosol (*n* = 6) or intradermal injection (*n* = 6) at study week zero or were left as unvaccinated controls (*n* = 7). All animals received ultra-low dose (ULD) aerosol challenge with *M. tuberculosis* Erdman strain (ERD ≤ 10 CFU) at study week 21 and were monitored for up to 16 weeks following infection (study week 37). Blue shaded circles represent procedures involving blood sample collection and application of immunological analyses, large open circles represent key study events: vaccination and ULD aerosol *M. tuberculosis* challenge, open circles indicate application of in vivo CT scanning. All animals were euthanized, and post-mortem (PM) necropsies conducted upon completion of the study schedule (shaded circles).

The experimental design and schedule of procedures, including the application of vaccination and experimental challenge, are summarized in Figure 1.

### 2.2. Vaccination

BCG vaccinations were delivered to sedated animals either as a 100-μL intradermal (ID) injection using Danish strain 1331 (SSI, Copenhagen, Denmark) delivered to the upper left arm, or by exposure to aerosolised BCG Danish strain 1331 created using an Omron MicroAir mesh nebuliser (Omron Healthcare UK Ltd., Milton Keynes, UK) and a modified paediatric anaesthesia mask. The vaccination dose was selected to be equivalent to a standard adult intradermal dose, after taking into account the expected losses in viable BCG titre associated with the aerosol delivery process [19]. BCG vaccine was prepared by adding 1 mL PBS to each vaccine vial to give an estimated concentration of 2 × 10^6^ to 8 × 10^6^ CFU/mL. Multiple vials were pooled together to ensure standardisation between vaccinations, before delivering one ml of the BCG preparation to each animal. The BCG titre in aerosol and ID BCG vaccine preparations was confirmed to be within the range specified by the manufacturer by bacterial culture on Middlebrook 7H11 selective agar containing oleic acid, bovine albumin, dextrose and catalase (OADC) (Biomerieux, Basingstoke, UK), for enumeration of viable colony forming units (CFU).

### 2.3. M. Tuberculosis Challenge Strain

The Erdman K01 stock (HPA-Sept 2011) used for challenge was prepared from stocks of the *M. tuberculosis* Erdman strain K 01 (BEI Resources). A stock suspension was initially prepared from a 5-mL bacterial starter culture originally generated from colonies grown on Middlebrook 7H11 supplemented with oleic acid, albumin, dextrose and catalase (OADC) selective agar (BioMerieux, Basingstoke, UK). A liquid batch culture was then grown to logarithmic growth phase in 7H9 medium (Sigma-Aldrich, Gillingham, UK) supplemented with 0.05% (*v/v*) Tween 80 (Sigma-Aldrich, Gillingham, UK). Aliquots were stored at −80 °C. The titre (viable bacilli per mL) of the stock suspension was determined from thawed aliquots by enumeration of colony-forming units following a culture of serial dilutions on Middlebrook 7H11 OADC selective agar.

### 2.4. Aerosol Exposure

#### 2.4.1. Apparatus and Procedure

The methodology and apparatus used to deliver *M. tuberculosis* via the aerosol route were as previously described [3,20,21]. In brief, aerosols were generated from a suspension of *M. tuberculosis* at a pre-determined concentration (see below) using a 3-jet Collison nebuliser (BGI, Butler, New Jersey, US) and delivered, using a modified Henderson apparatus [22] controlled by an AeroMP (Biaera Technologies, Hagerstown, Maryland, USA) control unit [23], to the nares of each sedated animal via a modified veterinary anaesthesia mask. A “head-out”, plethysmography chamber (Buxco, Wilmington, North Carolina, USA) enabled the aerosol to be delivered simultaneously with the measurement of the respiration rate.

#### 2.4.2. Quantification of Ultra-Low Aerosol Dose

The number of bacilli deposited and retained in the lungs of macaques cannot be measured directly, and, therefore, the dose is derived from a calculation based upon the concentration of viable organisms in the circulating aerosol and the volume of aerosol inhaled by the animal. This is termed the presented dose, and the derivation of this and the retained dose (the number of organisms assumed to be retained in the lung) have been described previously for high, medium and ultra-low aerosol doses [3,6,21]. Aerosol challenge data from previous experiments were used to predict the concentration of bacteria in the nebuliser required to result in a retained dose of approximately five viable bacilli [3,6,20,24].

### 2.5. Computed Tomography (CT) Imaging

CT scans were collected from animals using a 16-slice Lightspeed CT scanner (General Electric Healthcare, Milwaukee, WI, USA) at 3, 8, 11 and 16 weeks after aerosol exposure to *M. tuberculosis* (Figure 1), as described previously [25,26]. To enhance visualisation of lesions and lymph nodes, Niopam 300 (Bracco, Milan, Italy), a non-ionic, iodinated contrast medium, was administered intravenously (IV) at 2 mL/kg body weight. Scans were evaluated for the number and distribution of pulmonary lesions across lung lobes and the presence of nodule cavitation, conglomeration, consolidation as an indicator of alveolar pneumonia and a “tree-in–bud” pattern as an indicator of bronchocentric pneumonia. The lung-associated lymph nodes were assessed for enlargement and the presence of necrosis. Total lung volumes were calculated for each macaque using the COPD application of Philips IntelliSpace Portal software 9.0 (Phillips Healthcare, UK). Tuberculous lesions were identified and categorised as micronodules (spherical lesions smaller than 0.3 cm^3^) or regions of consolidation (pneumonic TB lesions bigger than 0.3 cm^3^). The volume of the consolidated regions was obtained using the Philips IntelliSpace Portal, Tumor Tracking application, and micronodule diameter was approximated as 1, 2, 3 or 4 mm. The total volume of damaged tissue was quantified by summing consolidated and micronodule volumes, and this was expressed as a percentage of the total lung volume measured for each macaque.

### 2.6. Clinical Assessment

Animals were sedated at two weekly intervals for blood sample collection and to measure body weight and temperature, red blood cell (RBC) haemoglobin levels and erythrocyte sedimentation rate (ESR). RBC haemoglobin levels monitored as an indication of anaemia, measured using a HaemaCue haemoglobinometer (Haemacue Ltd., Dronfield, UK). ESR was monitored using the Sediplast system (Guest Medical, Edenbridge, UK) as a general measure of *M. tuberculosis*-induced inflammation. Animal behaviour was observed throughout the study for contra-indicators, and the time of necropsy, if prior to the end of the planned study period, was determined by experienced primatology staff based on a combination of the following adverse indicators: depression or withdrawn behaviour, abnormal respiration (dyspnoea), loss of 20% of peak post-challenge weight, ESR levels elevated above normal (>20 mm), haemoglobin level below normal limits (<100 g/dL), increased temperature (>41 °C) and abnormal thoracic radiograph.

### 2.7. Immune Response Analysis

#### 2.7.1. Interferon-Gamma (IFN-γ) ELISpot

Interferon-gamma (IFN-γ) ELISpot assays were performed on peripheral blood mononuclear cells isolated from heparin anti-coagulated blood using standard methods, as previously described [24].

#### 2.7.2. Quantification of Secreted Biomarkers in Mycobacterial Antigen Stimulated Blood Cultures

Antigen-specific secretion of a range of cytokines, chemokines and growth factors was assessed by dilution of heparinised blood samples 1:10 with serum-free Roswell Park Memorial Institute (RPMI; R0) medium, before six day culture at 37 °C, 5% CO_2_, with 10 µg/mL tuberculin PPD (Statens Serum Institute, Copenhagen, Denmark), or 10 µg/mL phytohaemmagglutinin (PHA) from *Phaseolus vulgaris* (Sigma Aldrich, Gillingham, UK) as a positive control, or R0 alone as a negative control [27]. Following incubation, culture supernatants were aspirated using a 1-mL syringe (BD Biosciences, Oxford, UK) and passed through two 0.2-µm PES filters (GE Life Sciences, Amersham, UK), before storage at −80 °C. Secreted biomarkers in filtered culture supernatants were quantified using a 37-plex Procartaplex bead array assay (Thermo Fischer Scientific, Loughborough, UK), applied according to the manufacturer’s instructions, for detection of the following analytes: Eotaxin, G-CSF, GM-CSF, IFN-alpha, IFN-gamma, TNF-alpha, IL-10, IL-12p70, IL-13, IL-15, IL-17A, IL-18, IL-1b, IL-1RA, IL-2, IL-23, IL-4, IL-5, IL-6, IL-7, IL-8, IP-10, I-TAC (CXCL11), MCP-1 (CCL2), MIP-1alpha, MIP-1beta (CCL4), SDF-1alpha (CXCL12), MIG (CXCL9), CD40-Ligand, BLC (CXCL13), BDNF, SCF, VEGF-D, bNGF, FGF-2, PDGF-BB, VEGF-A. Culture filtrates were analysed using a Luminex Magpix instrument (Luminex Corporation, Hertogenbosch, Netherlands) equipped with the xPONENT 4.2 software package. Standard curves were generated in duplicate and used to interpolate the concentration of each analyte. All data below the limit of detection specified by the kit manufacturer was assigned a zero value. The analyte concentration measured in negative control cultures was subtracted from antigen-stimulated samples to obtain a measure of antigen-specific biomarker secretion, and the analyte concentration was multiplied by ten to account for the initial dilution of the sample and provide a value per ml of blood.

### 2.8. Intracellular Cytokine Staining and Memory T-cell Analysis

#### 2.8.1. Polyfunctional Intracellular Cytokine Staining and Antigen-Specific Memory T-cell Assay

Intracellular cytokine staining (ICS) was performed using 1 × 10^6^ PBMC in medium (R10) consisting of RPMI 1640 supplemented with L-glutamine (2 mM), penicillin (50 U/mL) and streptomycin (50 μg/mL) (all from Sigma Aldrich, Gillingham, UK) and 10% heat-inactivated foetal bovine serum (Labtech Ltd., Uckfield, UK). These cells were stimulated with a 10-μg/mL solution of CD28 and CD49d co-stimulatory antibodies (both from BD Biosciences, Oxford, UK) and either 10 μg/mL PPD (SSI, Copenhagen, Denmark), or 5 μg/mL staphylococcal enterotoxin b (SEB) (Sigma Aldrich, Gillingham, UK), or R10 medium alone as a negative control for a total of six hours at 37 °C in a 5% CO_2_ supplemented incubator. Following the initial two hours of incubation, the protein transport inhibitor Brefeldin-A (Sigma Aldrich, Gillingham, UK) was added to the incubation mixture at a final concentration of 10 μg/mL. Following incubation, cells were washed with FACS buffer consisting of PBS + 1% FCS and incubated for 30 min at room temperature with optimal dilutions of the amine-reactive Live/Dead Fixable Red viability cell stain (Life Technologies, Renfrew, UK) and the antibodies CD4 APC-H7, CD8 PerCP-Cy5.5, CD95 Pe-Cy7 (all from BD Biosciences, Oxford, UK), CD28 BV-421, CD45RA-PeCy7 (Biolegend, London, UK), CCR7-PE (eBioscience, Hatfield, UK), CD14-ECD and CD20-ECD (both from Beckman Coutler, High Wycombe, UK). Following surface marker staining, the cells were washed and then permeabilised by incubation at room temperature for 15 min with Fix/Perm reagent (BD Biosciences, Oxford, UK). Further cell washes were applied using Permwash buffer (BD Biosciences, Oxford, UK), before staining for intracellular antigens by incubation at room temperature for 30 min with the antibodies CD3-AF700, IFN-γ-FITC, TNF-α-BUV395 (all from BD Biosciences, Oxford, UK), IL-2-APC (Miltenyi Biotech Ltd., Bisley, UK), IL-17-BV711 (Biolegend, London, UK). BD Compbeads (BD Biosciences, Oxford, UK) were labelled with the above fluorochromes for use as compensation controls. Following antibody labelling, cells and beads were washed by centrifugation and fixed in 4% paraformaldehyde solution (Sigma Aldrich, Gillingham, UK) prior to flow cytometric acquisition.

#### 2.8.2. Flow Cytometric Acquisition and Analysis

Cells were analysed using a five laser LSRII Fortessa instrument (BD Biosciences, Oxford, UK), and data were analysed using FlowJo (version 9.7.6, Treestar, Ashland, US). Cytokine-producing T-cells were identified using a forward scatter-height (FSC-H) versus side scatter-area (SSC-A) dot plot to identify the lymphocyte population, to which appropriate gating strategies were applied to exclude doublet events, non-viable cells, monocytes (CD14^+^) and B cells (CD20^+^). For ICS analysis, sequential gating through CD3^+^, followed by CD4^+^ or CD8^+^ gates, were used before individual cytokine gates to identify IFN-γ-, IL-2-, TNF-α- and IL-17-producing populations. Polyfunctional cells were identified using Boolean gating combinations of individual cytokine-producing CD4 or CD8 T-cells. Antigen-specific T-cell memory profiles were identified by applying a summed CD4 or CD8 cytokine Boolean combination, followed by gating for CD95 surface staining. Differentiation of effector, transitional effector, central memory, and stem cell memory T-cell populations was established by CD45RA, CD28 and CCR7 expression patterns (Section 3.9.3). The software package PESTLE version 1.7 (Mario Roederer, Vaccine Research Centre, NIAID, NIH) was used for background subtraction to obtain antigen-specific polyfunctional ICS and memory T-cell cytokine responses. Graphpad Prism (version 8.0.1) was used to generate graphical representations of flow cytometry data.

### 2.9. Necropsy

Animals were anaesthetised and clinical data collected. Blood samples were taken prior to euthanasia by intracardiac injection of a lethal dose of anaesthetic (Dolelethal, Vétoquinol UK Ltd., Towcester, UK 140 mg/kg). A post-mortem examination was performed immediately and gross pathological changes were scored using an established system based on the number and extent of lesions present in the lungs, spleen, liver, kidney and lymph nodes, as described previously [3]. Samples of spleen, liver, kidneys and tracheobronchial, inguinal and axillary lymph nodes were removed and sampled for quantitative bacteriology. The lungs, including the heart and lung-associated lymph nodes, were removed intact. The lymph nodes were measured and examined for lesions. The whole lung was fixed by intra-tracheal infusion with 10% neutral buffered formalin (NBF) using a syringe and 13CH Nelaton catheter (J.A.K. Marketing, York, UK). To ensure complete and rapid fixation of the tissue, the catheter tip was inserted through the trachea into each main-stem bronchus in turn and lobes on each side were infused until they were expanded to a size consistent with normal inspiratory dimensions; the trachea was then ligated to retain the fluid. The infused lung was immersed in 10% NBF. In addition, samples of kidneys, liver, spleen, and sub-clavicular, hepatic inguinal and axillary lymph nodes were fixed in 10% NBF.

### 2.10. Pathology Studies

The fixed lungs were sliced serially, and each lung lobe evaluated separately and scored based on the number and extent of lesions present by application of the pathology scoring system applied at necropsy [25]. Discrete lesions were counted in the parenchyma, and where lesions had coalesced, these were measured and recorded. Areas of consolidation in each lobe were recorded and quantified. The remaining tissues were examined during trimming.

### 2.11. Histological Examination

Representative samples from each lung lobe and other organs were processed to paraffin wax, sectioned at 3–5 µm and stained with haematoxylin and eosin (HE). For each lung lobe, tissue slices containing macroscopically visible lesions were chosen for histological examination. Where gross lesions were not visible, a sample was taken from a pre-defined anatomical location from each lobe to establish consistency between animals. The nature and severity of the microscopic tuberculous lesions were evaluated subjectively by a pathologist who was blinded to prevent bias, and lesions were graded according to organisation state and severity as previously described [28]. Furthermore, additional features of disease were recorded as present or absent; these included multi-nucleated giant cells, airway invasion, fulminating pneumonia (the latter defined as representing inflammatory changes with the parenchyma which extended between granulomas) and the presence of lesions that appeared to originate directly from within the bronchus-associated lymphoid tissue (BALT).

### 2.12. Bacteriology

The spleen, kidneys, liver and tracheobronchial lymph nodes were sampled for the presence of viable *M. tuberculosis* post-mortem [20]. Where available, tissue sections with and without visible tuberculous lesions were collected for analysis.

Weighed tissue samples were homogenised in 2 mL of sterile water, and either serially diluted in sterile water prior to being plated, or plated without dilution onto Middlebrook 7H11 OADC selective agar. Plates were incubated for three weeks at 37 °C, and resultant colonies were confirmed as *M. tuberculosis* and counted. Mean colony-forming units (CFU) per gram from each tissue sample were determined.

### 2.13. Statistical Analyses

Differences in measures of the immune response, including IFN-γ ELISpot and biomarker secretion profiles, T-cell functional and memory population frequencies measured by polyfunctional flow cytometry, as well as pathology scores, pulmonary disease measures and clinical measures of disease burden recorded at the end of the study, were conducted using the non-parametric Mann–Whitney U-test function in GraphPad Prism version 6.05 (GraphPad Software Inc, La Jolla, California, USA). Similarly, GraphPad was used to calculate the area under the curve (AUC) of each animal’s response for further comparison of the cell-mediated response measured by IFN-γ ELISpot between the groups. Negative values in antigen-specific ICS and IFN-γ ELISpot data generated by background subtraction were replaced by a zero value [29]. Differences in the rate of disease progression (survival rates) of animals in each test group were compared with a Mantel–Cox log rank test and the Gehan–Breslow–Wilcoxon test in GraphPad Prism, version 6.05.

## 3. Results

### 3.1. Safety of ID and Aerosol Delivered BCG

In agreement with previous reports [3,19], BCG vaccinations were well tolerated by all animals. Mild local reactions, including induration and erythema at the site of immunization, were observed following ID vaccination; adverse indicators were not apparent in animals that received the aerosol BCG vaccination regimen. Clinical measures, including body weight, temperature, peripheral lymph node size, red cell haemoglobin concentration (Hb), and erythrocyte sedimentation rate, remained within normal ranges following aerosol or ID BCG vaccination.

### 3.2. M. Tuberculosis Challenge Dose and Disease Progression

**Figure 2:**
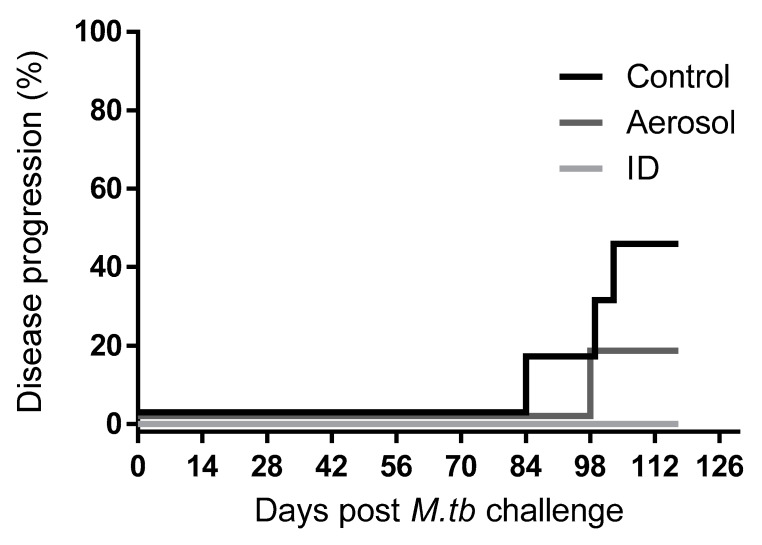
Kaplan–Meier plot indicating proportions of vaccinated and unvaccinated non-human primates (NHPs) able to control disease progression following ultra-low dose aerosol challenge with *M. tuberculosis*.

Animals were challenged with a median estimated retained dose of three CFU aerosolised *M. tuberculosis* Erdman Strain K01 (Table 1). Changes in clinical parameters, including weight loss and perturbations in temperature and Hb concentration, were observed across all vaccination groups within four weeks of challenge. Four animals were euthanised prior to completion of the 16-week post-*M. tuberculosis* challenge schedule due to weight and behavioural changes indicative of progressive disease which met humane endpoint criteria; these included three unvaccinated control animals and one aerosol BCG-vaccinated animal (Figure 2). One ID BCG-vaccinated animal was removed from the study three weeks following *M. tuberculosis* challenge due to a non-disease related injury. Clinical and disease parameters recorded from this animal were excluded from the analysis to avoid biasing of efficacy readouts. The time at which animals progressed to meet predefined humane end-point criteria was compared between the vaccination groups using a log-rank or Gehan–Breslow–Wilcoxon test (Figure 2); these analyses did not indicate significant differences between the vaccination regimens (*p* = 0.18 and 0.20).

### 3.3. In Life CT Evaluation of Disease Pathology

**Figure 3:**
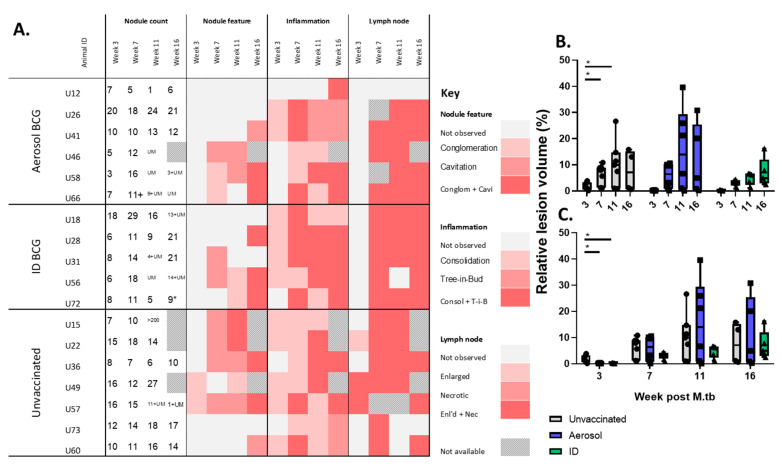
Development of thoracic pathology measured by computed tomography (CT) X-ray scanning at regular intervals following low dose aerosol challenge with *M. tuberculosis*. (**A**) Pulmonary nodule counts recorded from thoracic CT scans of individual animals at weeks 3, 7, 11 and 16 following aerosol challenge with *M. tuberculosis* are shown; UM = unmeasurable due to lesion conglomeration and lung lobe consolidation. Colour gradients in heat map plots represent the presence or absence of typical pulmonary features of tuberculous disease, including nodule conglomeration and cavitation, consolidation and tree-in-bud morphology due to pulmonary inflammation, and lymph node enlargement or necrosis. (**B**,**C**) Relative volume of tuberculous lung lesions quantified by CT analysis. Data points show the percentage area of damaged lung tissue measured in individual animals, with group median, interquartile range and minimum and maximum values shown by box and whisker plots. Asterisks denote significant differences measured within (Wilcoxon, Figure B) and between treatment groups (Mann–Whitney, Figure C), * *p* ≤ 0.05.

CT imaging was applied at regular intervals following exposure to low dose aerosols of *M. tuberculosis* for the monitoring of disease burden and assessment of features characteristic of TB disease pathology. Three weeks after aerosol challenge with *M. tuberculosis*, more nodules were counted in the lungs of unvaccinated macaques (group median 12) in comparison to the groups that received BCG intradermally (median 8) or by aerosol (median 7) (Figure 3A). A comparison of the early lesions quantified in unvaccinated animals to the reduced number of lesions measured in the vaccinated groups is indicative of an initial vaccine effect in both the aerosol- and ID-vaccinated animals. This initial vaccine-induced reduction of disease was also apparent when the volume of lung tissue affected by tuberculous pathology was quantified in relation to total pulmonary volume. This revealed a significant reduction in the proportion of the lung affected by the disease in the ID and aerosol BCG-vaccinated groups three weeks after *M. tuberculosis* infection (Figure 3B,C). The occurrence of further features typical of *M. tuberculosis*-related pulmonary disease, such as areas of pneumonia associated with the lung parenchyma (consolidation) and airways (tree-in-bud morphology), were apparent in animals from all treatment groups from three weeks post-*M. tuberculosis*, progressing to include cavitation from seven weeks after *M. tuberculosis* exposure (Figure 3A).

### 3.4. Disease Pathology Recorded in Pulmonary and Extra-Pulmonary Tissues

**Figure 4:**
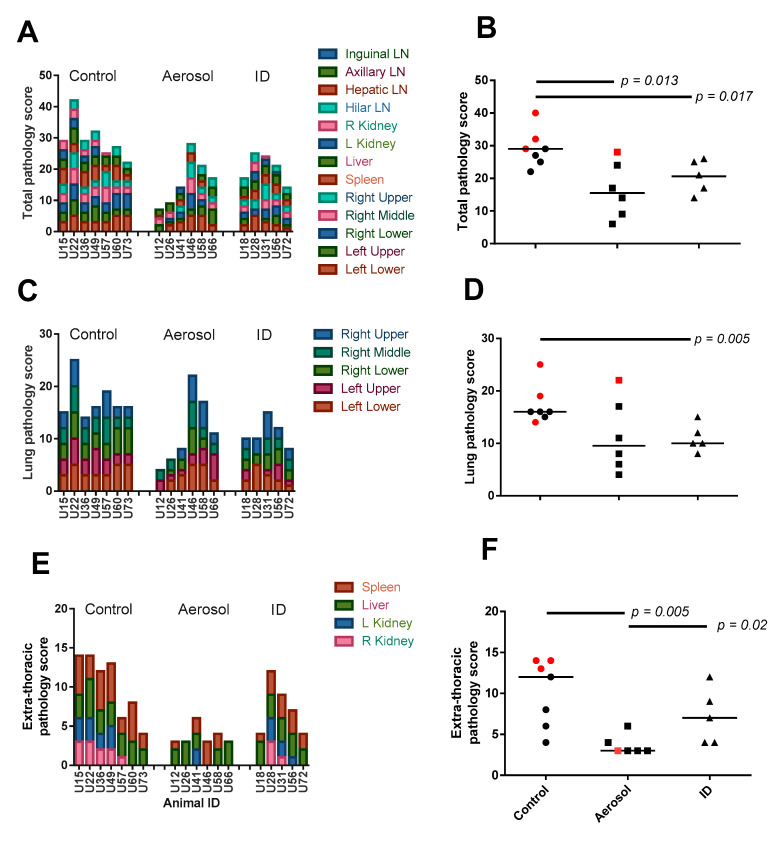
Gross-pathology assessed at necropsy. The occurrence and extent of tuberculous disease pathology were recorded at necropsy using a standardised scoring system. This approach was used to determine total pathology scores from a range of pulmonary and extra-pulmonary tissues (**A**,**B**), lung pathology derived from scores assessed in each lung lobe (**C**,**D**), and an extra-thoracic score comprised of scores recorded from tissues typically associated with bacterial dissemination (**E**,**F**). Plots **A**, **C** and **E** show scores recorded from specific tissues in each individual animal, whereas plots **B**, **D** and **F** display summed tissue scores with group median values indicated. Animals that met humane end-point criteria are indicated with red data points. Significant differences between groups determined by Mann–Whitney U-test are shown.

The extent of tuberculosis disease pathology was recorded at necropsy in a range of pulmonary, lymphoid and extra-thoracic organs using a scoring system based on the number of lesions and extent of disease [20]. Lung pathology was assessed in formalin-fixed tissue sections prepared from each lung lobe. Total pathology scores were established by summing scores recorded in each tissue, whereas pulmonary and dissemination scores were derived from summed lung lobe scores or summed scores recorded from extra-thoracic tissues excluding lymph nodes (Figure 4A–F). A comparison of group median total pathology scores revealed a significant reduction in tuberculosis pathology in both aerosol and ID BCG vaccination groups relative to the unvaccinated control group (*p* ≤ 0.017). Lung pathology was also reduced, reaching significance in ID BCG-vaccinated animals (*p* = 0.005), and disease pathology in extra-thoracic tissues was significantly lower in aerosol-vaccinated animals relative to both unvaccinated (*p* = 0.005) and ID BCG-vaccinated animals (*p* = 0.02), indicating that aerosol BCG vaccination had reduced the occurrence of *M. tuberculosis*-attributable disease pathology in sites typically associated with extrapulmonary *M. tuberculosis* dissemination.

### 3.5. Histopathology

**Figure 5:**
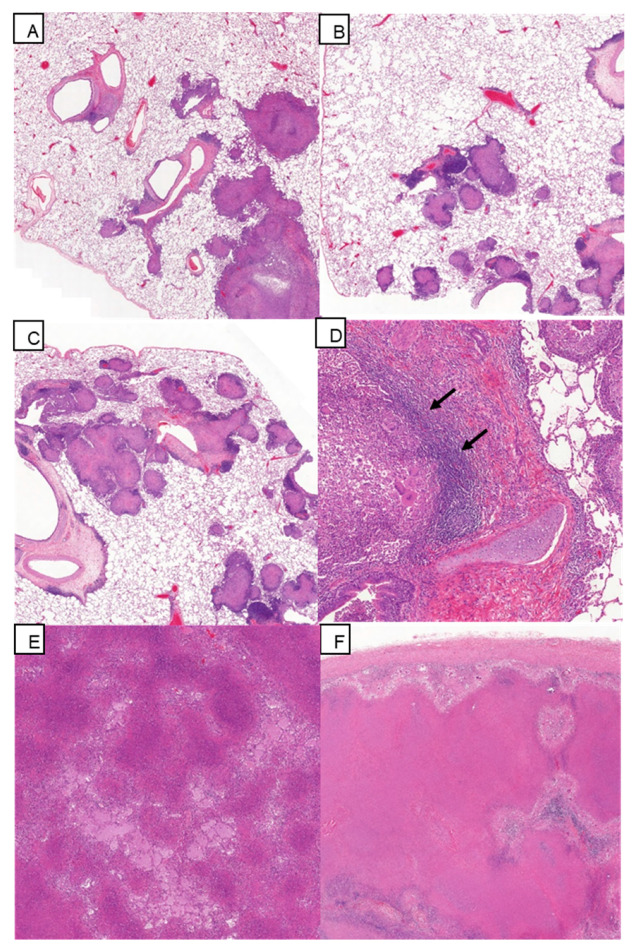
Microscopic changes in the lung and associated lymph node. (**A**) Lung, Animal U41 (Group A). (**B**) Lung, Animal U18 (Group B). (**C**) Lung, Animal U36 (Group C). Multifocal, tuberculous lesions of varying types scattered throughout the parenchyma. (HE, 2× objective). (**D**) Lung, Animal U58 (Group A). Bronchus-associated lymphoid tissue invaded by tuberculous granuloma, with a peripheral rim of lymphoid tissue remaining (arrows) (HE, 10× objective). (**E**) Lung, Animal U46 (Group A). Fulminating pneumonia. (HE, 5× objective). (**F**) Lung-associated lymph node, Animal U26 (Group A). Effacement of the lymphoid tissue by caseous necrosis and granulomatous inflammation. (HE, 2× objective).

The occurrence of granulomatous, microscopic lesions in tissues examined is summarised in Table 2. In the lung, a range of lesion types was present in each animal from all groups (Figure 5A–C), with the most frequently observed lesions being types 4 to 6. There did not appear to be prominent differences in either lesion type or their frequency of occurrence between the groups. The presence of lesions appearing to originate in BALT was observed with similar frequency between all groups (Figure 5D); this was also true for the presence of multi-nucleated giant cells within lesions. Fulminating pneumonia (Figure 5E) was noted in a proportion of animals in all groups, and features such as airway invasion and cavitation were noted in all groups but with a greater frequency in unvaccinated animals. In addition, the local spread of granulomatous lesions to the pleura covering the rib cage was present in all animals of the unvaccinated group, compared to three aerosol BCG-vaccinated and three ID BCG-vaccinated animals. The lung-associated lymph nodes of all animals in all groups were similar, with large areas of parenchyma replaced by granulomatous inflammation including widespread caseous necrosis (Figure 5F), whereas, microscopic evidence of tuberculous pathology that was present in the peripheral lymph nodes (axillary, inguinal, hepatic and sub-clavicular) of unvaccinated and ID BCG-vaccinated animals, was not observed in the aerosol-vaccinated group.

### 3.6. Viable M. Tuberculosis Recovered from Extra-Pulmonary Tissues

**Figure 6:**
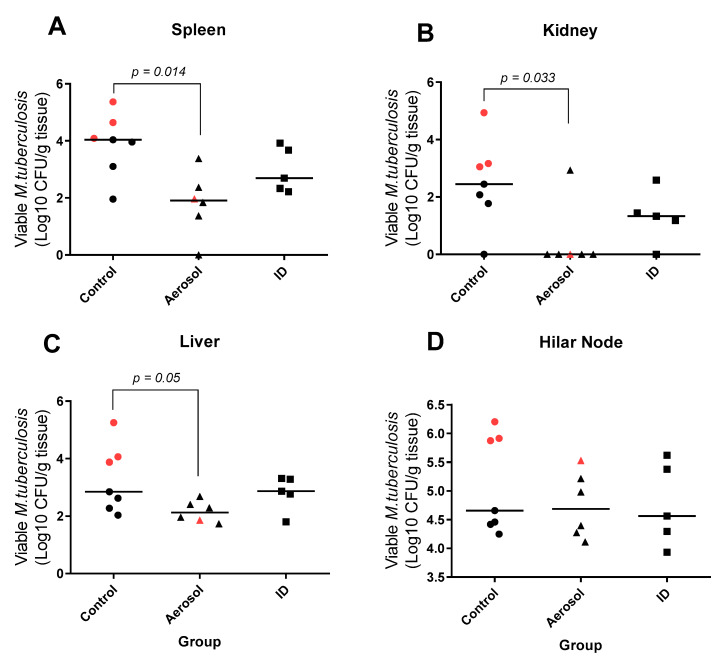
Viable *M. tuberculosis* recovered from Hilar lymph nodes and extra-thoracic tissues. Representative tissue samples collected from spleen (**A**), kidney (**B**), liver (**C**), and tracheobronchial lymph nodes (**D**) were homogenised and cultured on selective agarose for assessment of *M. tuberculosis* bacterial load in extrapulmonary and extra-thoracic tissues. Individual points represent log_10_CFU/g tissue recovered from each animal with the group median indicated by horizontal bar. Animals in which disease met humane end-point criteria are indicated with red data points. Significant differences between groups determined by Mann–Whitney U-test are shown.

Tissue samples were collected from a range of extra-pulmonary tissues, including the spleen, kidneys, liver and tracheobronchial (hilar) lymph nodes, for the quantification of viable *M. tuberculosis* by bacterial culture (Figure 6). Extra-pulmonary dissemination of *M. tuberculosis* was detected in all animals with an equivalent bacterial load measured in the tracheobronchial lymph nodes, a primary site of dissemination from the lung, regardless of vaccination status (Figure 6D). However, significant differences in the quantity of viable bacteria recovered from extra-thoracic tissues were observed between the treatment groups, with *M. tuberculosis* CFU/g of spleen, liver and kidneys significantly lower in aerosol BCG-vaccinated animals in comparison to the unvaccinated group. In contrast, the bacterial burden measured in tissue samples collected from the ID BCG-vaccinated group did not differ significantly from that measured in unvaccinated animals (Figure 6B–D).

### 3.7. Frequency of Antigen-Specific IFN-γ Secreting Cells Measured by ELISpot

**Figure 7:**
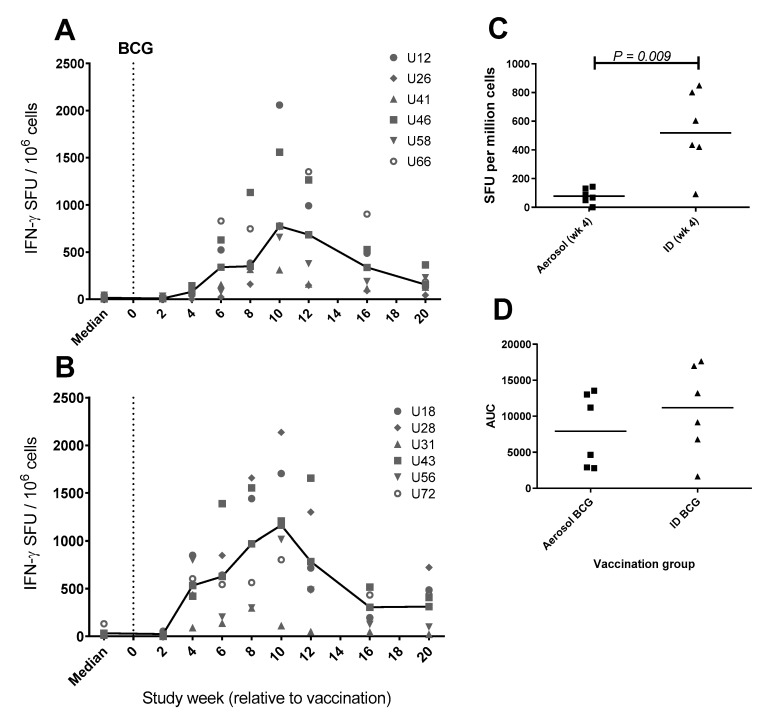
Systemic purified protein derivative (PPD)-specific IFN-γ spot forming unit (SFU) frequency measured by ELISpot assay following aerosol or ID Bacille Calmette-Guérin (BCG) vaccination. PPD-specific IFN-γ SFU measured in individual aerosol (**A**) or ID (**B**) BCG-vaccinated animals are shown as points with vaccination group median line. Vaccination with aerosol or intradermal BCG is indicated by a dotted line at study week zero. Pre-vaccination SFU frequency is shown as the median of at least 3 replicate values for each animal. Plot **C** shows a dot plot comparison of IFN-γ SFU frequency four weeks after vaccination, and plot **D** shows area under the curve (AUC) values calculated from each animal’s SFU frequency profile between weeks 0–20. The group median is indicated by horizontal lines and Mann–Whitney U-test significance values are shown.

Systemic immune responses induced by aerosol or ID BCG vaccination and ULD *M. tuberculosis* challenge were profiled using an ex vivo IFN-γ ELISpot assay. Vaccination-induced PPD-specific IFN-γ spot forming units (SFU) were detected four weeks following ID and six weeks after aerosol BCG vaccination (Figure 7), indicating delayed initiation of the systemic adaptive immune response following aerosol vaccination, as has previously been described [19]. Comparison of IFN-γ SFU’s between vaccination groups revealed that the frequency of IFN-γ secreting cells was significantly lower in the aerosol BCG-vaccinated group at the four-week time point (*p* = 0.009; Figure 7C). Nevertheless, IFN-γ SFU frequencies peaked at comparable levels ten weeks post-vaccination in both the aerosol and ID BCG-vaccinated groups and remained comparable for the rest of the pre-*M. tuberculosis* challenge period of the study. Indeed, the area under the curve analysis (AUC) of the IFN-γ SFU vaccination phase indicated equivalence between the aerosol and ID BCG-induced response (*p* = 0.49; Figure 7D), and comparative analysis applied one week prior to *M. tuberculosis* challenge (study week 20), indicated that there was no significant difference between the IFN-γ SFU frequency measured in the vaccination groups at the final pre-challenge time point.

**Figure 8:**
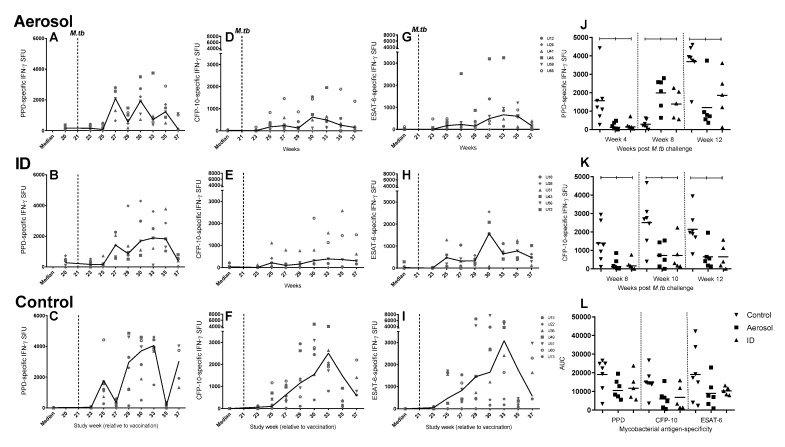
Systemic PPD-, CFP10- and ESAT-6-specific IFN-γ spot forming unit frequency measured by ELISpot assay following aerosol challenge with *M. tuberculosis*. Mycobacterial antigen-specific IFN-γ SFU measured in individual aerosol (**A**,**D**,**G**), or ID (**B**,**E**,**H**) BCG-vaccinated and unvaccinated control (**C**,**F**,**I**) animals during the post-challenge phase of the study (weeks 21–37) are shown as points with the vaccination group median line plotted. Ultra-low dose (ULD) aerosol *M. tuberculosis* challenge is indicated by a dotted line at study week 21. Plots **J** and **K** show PPD- (**J**) and CFP-10-specific (**K**) IFN-γ SFU frequencies between vaccinated and unvaccinated groups at specific weeks post-*M. tuberculosis* exposure, where significant differences were measured by Mann–Whitney U-test (*p* ≤ 0.05*)*. Plot **L** shows AUC values calculated for each vaccination group from the PPD-, CFP-10- and ESAT-6-specific response profiles following *M. tuberculosis* challenge. Group medians are indicated by horizontal lines and Mann–Whitney U-test significance is indicated by floating bars.

Ultra-low dose *M. tuberculosis* challenge led to increases in *M. tuberculosis*-specific IFN-γ SFU frequencies, measured in response to stimulation with PPD or peptides spanning the CFP-10 or ESAT-6 sequence, in all animals (Figure 8A–I). Comparison of IFN-γ SFU frequencies between the unvaccinated control and vaccinated groups revealed significantly greater PPD-specific IFN-γ cell frequencies in unvaccinated animals, 4 and 10 weeks following *M. tuberculosis* challenge (*p* = 0.002 and *p* = 0.003), and in CFP-10-specific IFN-γ SFU frequencies at weeks 8, 10 and 12 (*p* = 0.015, *p* = 0.015 and *p* = 0.004) post-challenge (Figure 8J,K). Significant differences were not detected between the experimental groups in terms of ESAT-6–specific IFN-γ SFU profiles when responses were compared at individual study time points or across the post-*M. tuberculosis* challenge phase of the study (Figure 8L).

### 3.8. Mycobacterial Antigen-Specific Secretion of Biomarkers Measured by Cytokine Bead Array

**Figure 9:**
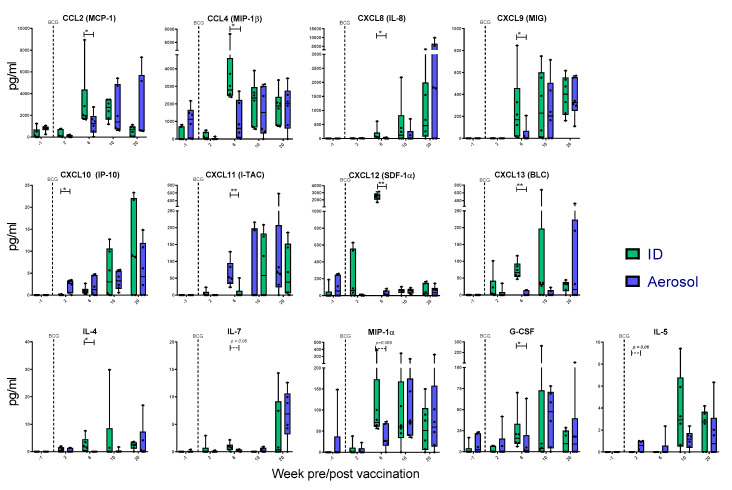
PPD-specific secretion of cytokine and chemokine biomarkers measured in whole blood culture supernatant. Box plots display the group median titre of each cytokine or chemokine +/- interquartile range measured prior to (-1) and at weeks 2, 4, 10 and 20 following vaccination. Dots represent titres measured in individual animals with the group minimum and maximum values shown by whiskers. Significant differences measured by Mann–Whitney U-test between the groups are indicated, * *p* ≤ 0.05; ** *p* ≤ 0.01.

The secretion of 37 cytokines and chemokines was quantified in PPD-stimulated whole blood cultures collected prior to, and at weeks 2, 6, 10 and 20 following aerosol or ID BCG vaccination, to explore potential differences in immunomodulatory signalling associated with the route of vaccine delivery. Of the 37 analytes measured, the concentration of 24 (Eotaxin, GM-CSF, IFN-alpha, IFN-gamma, TNF-alpha, IL-10, IL-12p70, IL-13, IL-15, IL-17A, IL-18, IL-1b, IL-1RA, IL-2, IL-23, IL-6, CD40-Ligand, BDNF, SCF, VEGF-D, bNGF, FGF-2, PDGF-BB, VEGF-A) was either undetectable (below the limit of detection of the assay system), or did not differ significantly following BCG vaccination by either route and was excluded from further analyses. Mycobacterial antigen-specific secretion of eight chemotactic signalling molecules (chemokines) IL-8 (CXCL8), IP-10 (CXCL10), I-TAC (CXCL11), MCP-1 (CCL2), MIP-1beta (CCL4), SDF-1alpha (CXCL12), MIG (CXCL9) and BLC (CXCL13), as well as the cytokines and growth factors IL-4, IL-5, IL-7, MIP-alpha and G-CSF, was found to increase significantly following vaccination by either aerosol or ID injection relative to pre-vaccination levels (*p* ≤ 0.05; Figure 9). In most cases, analyte concentrations measured in ID BCG-vaccinated animals were significantly higher than in the aerosol-vaccinated group six weeks after vaccination, although titres reached equivalence at later study weeks, indicating that it was the kinetics of the response that differed between the groups rather than its overall composition. Notably, the concentration of the chemokine IP-10, a known pro-inflammatory biomarker involved in the recruitment of pro-inflammatory cells during mycobacterial infection [30,31], and the cytokine IL-5, which is more closely associated with an anti-inflammatory Th2 response profile [32] and negatively modulates *M. tuberculosis*-specific CD4 T-cell TNF-α production in vitro [33], were detected at significantly (or close to significance, *p* = 0.06*,* for IL-5) higher concentrations in aerosol-vaccinated animals two weeks after vaccination.

### 3.9. Cellular Immune Responses Measured by Flow Cytometry

#### 3.9.1. CD4 and CD8 T-cell Functional Profiles Following Aerosol or ID BCG Vaccination

**Figure 10:**
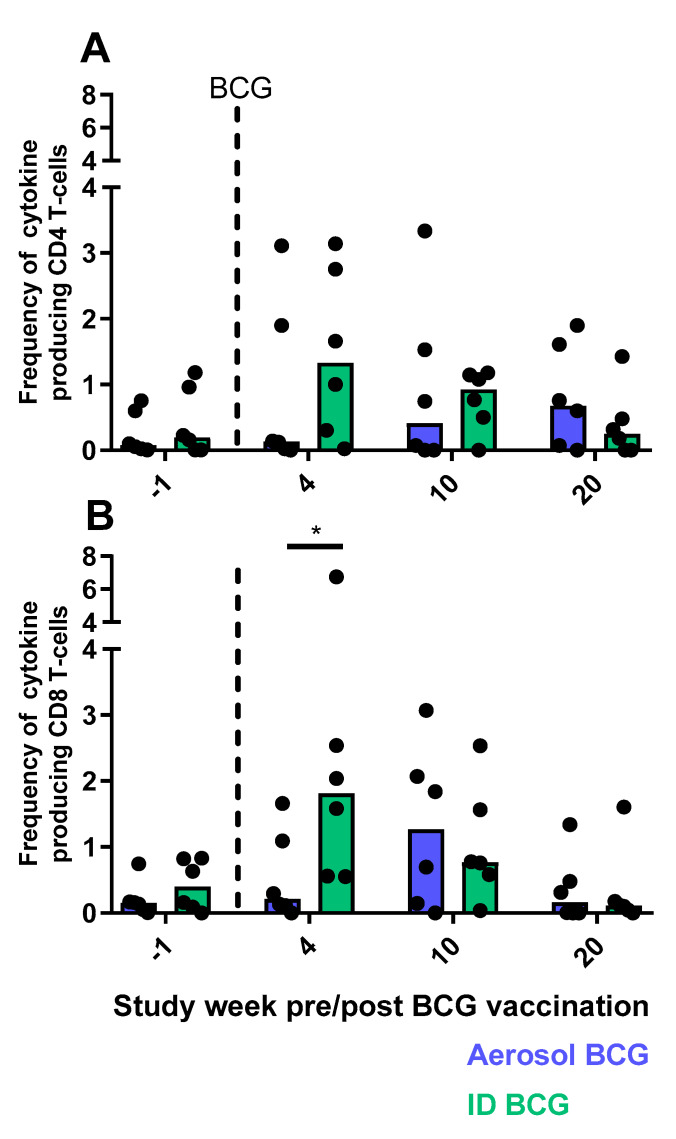
Total PPD specific-cytokine production by CD4 and CD8 T-cells. Dots represent the summed frequency of CD4 (**A**) and CD8 (**B**) T-cells producing IFN-γ, IL-2, TNF-α or IL-17 measured in peripheral blood mononuclear cells (PBMCs) collected from individual animals prior to (week -1) and following aerosol (blue bars) or intradermal (green bars) BCG. Bars show the group medians with significant differences determined by Mann–Whitney U-test indicated by an asterisk, *p* ≤ 0.05.

The frequency and functional profile of CD4 and CD8 T-cell populations were assessed by multi-parameter ICS staining to measure the production of the cytokines IFN-γ, TNFα, IL-2 and IL-17 at regular intervals during the vaccination phase of the study. Aerosol and ID BCG vaccination induced CD4 and CD8 T-cells with functional profiles typical of the Th1 (producing IFN-γ or TNFα) and Th17 (producing IL-17) phenotypes, as well as IL-2 producing populations (Figure S1). Despite these similarities in the functional repertoire, comparison of the total (summed) cytokine response revealed that vaccine-induced cytokine production was delayed following aerosol, relative to ID, BCG vaccination with peak frequencies of cytokine-producing CD4 and CD8 T-cells measured at the four-week post-vaccination time point in ID-vaccinated animals, whereas aerosol BCG vaccination led to peaks in CD8 T-cell cytokine production at week 10 and at week 20 for CD4 subsets (Figure 10).

#### 3.9.2. Multifunctional T-cell Populations Induced by Aerosol BCG or ID BCG Vaccination

**Figure 11:**
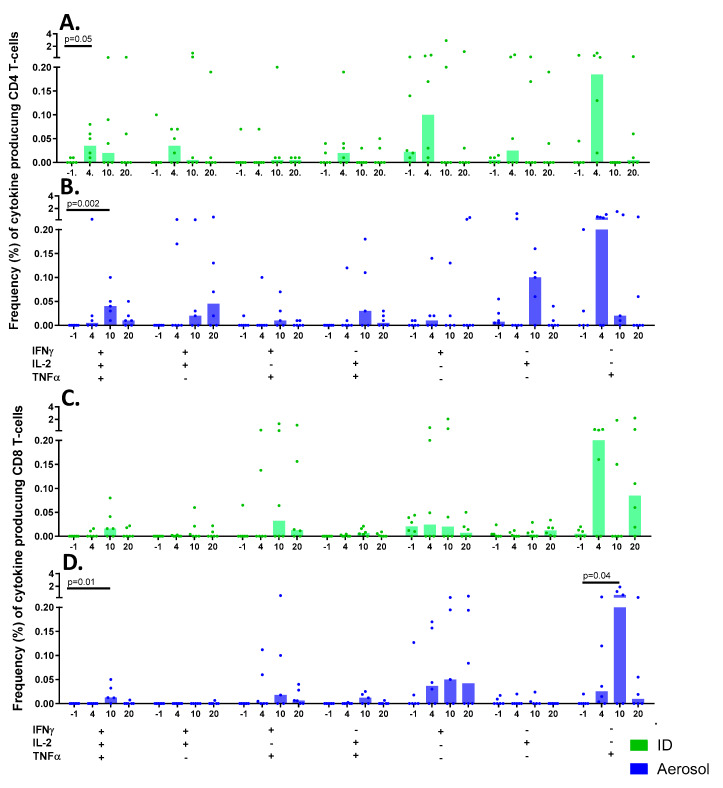
PPD-specific polyfunctional CD4 and CD8 T-cell profiles measured in PBMC. Bar charts show vaccination group median frequencies of cytokine-producing CD4 (**A**,**B**) and CD8 (**C**,**D**) T-cells measured in PBMCs at specific weeks prior to (week -1) and following (weeks 4, 10 and 20) BCG vaccination. Significant differences between pre- and post-vaccination values are indicated by bars vaccination with *p*-values shown. Cytokine frequencies measured in individual animals are represented by dots.

The functional profile of T-cell subsets was explored in greater detail by identification of cells producing multiple combinations of the cytokines IFN-γ, TNFα and IL-2 simultaneously in response to stimulation with tuberculin PPD. BCG vaccination delivered by aerosol or by ID injection led to significant increases in IFN-γ-, IL-2- and TNF-α-producing polyfunctional CD4 T-cells (*p* = 0.02*, p* = 0.05); and trends for increased IFN-γ and IL-2, and TNF-α and IL-2 multifunctional populations (Figure 11). CD4 T-cell populations producing IFN-γ alone, typically considered to indicate a more terminally differentiated phenotype [34], were more prevalent in the ID BCG-vaccinated group, whereas IL-2-producing monofunctional CD4 populations were more prevalent in aerosol BCG-vaccinated animals, potentially indicating greater proliferative capacity [34]. The frequency of polyfunctional CD8 T-cells increased significantly at 10 weeks following aerosol BCG vaccination (*p* = 0.01), whereas other cytokine-producing CD8 T-cell populations primarily consisted of cells producing IFN-γ or TNF-α alone (Figure 11C,D). Despite the reoccurring trend for the cellular immune response to be delayed when BCG was delivered as an aerosol, with peak cytokine production from CD4 and CD8 T-cell occurring at week 10 as opposed to week 4 following ID vaccination, significant differences were not measured between the vaccination groups.

#### 3.9.3. Antigen-Specific Memory T-cell Profiles Following Aerosol or ID BCG Vaccination

**Figure 12:**
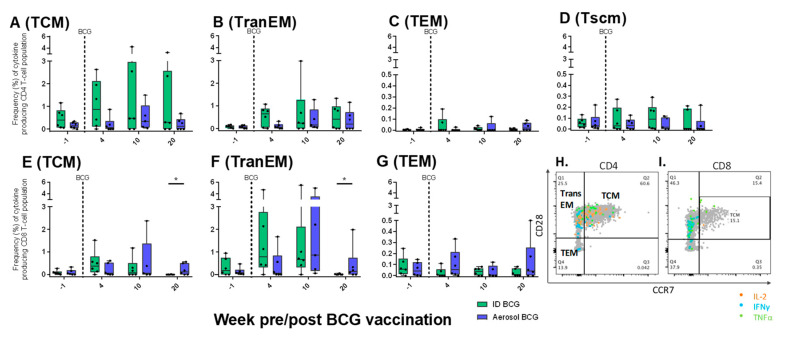
Antigen-specific CD4 and CD8 memory T-cell profiles measured in PBMCs. Plots H and I show representative bivariate density plots of central to effector memory T-cell populations defined by the pattern of CD28 and CCR7 staining on CD95 expressing CD4 (**H**) and CD8 (**I**) T-cells, overlaid with cytokine-producing cells represented as coloured dots. Plots **A**–**G** show the frequency of memory CD4 (Plots **A**–**D**) and CD8 (plots **E**–**G**) T-cell populations producing IFN-γ, IL-2 or TNF-α, measured prior to (week -1) and following (weeks 4, 10 and 20) BCG vaccination. Box plots show the group median and interquartile range with minimum and maximum values shown by whiskers. Significant differences measured by Mann–Whitney U-test are indicated by an asterisk (*), *p* ≤ 0.05.

To assess the memory status of cytokine-producing T-cell populations, central and effector memory T-cells were identified by expression of the cell activation markers CD95 and CD45RA, as well as differential expression patterns of the co-stimulatory receptor CD28 and lymph node homing marker CCR7. Therefore, T-cell memory phenotype was determined on CD95^+^ cells as CD28^+^CCR7^+^ central memory (TCM), CD28^+^CCR7^−^ transitional effector memory (TranEM), effector memory (TEM) CD28^-^CCR7^−^, and stem cell memory CD45RA^+^CD28^+^CCR7^+^ (Tscm) (Figure 12H,I) [35,36,37]. Using this classification, antigen-specific cytokine-producing memory CD4 and CD8 T-cell profiles were assessed and compared at regular intervals following aerosol and ID BCG vaccination (Figure 12A–G). Cytokine-producing CD4 T-cells primarily occupied TCM and TranEM phenotypes, with only low frequencies of cytokine-producing TEM cells detected. Antigen-specific CD8 Tscm populations were not detected, whereas cytokine-producing CD4 Tscm populations were detected at low frequency and remained unchanged from pre-vaccination levels, indicating that variation in the functional parameters measured is not induced by BCG vaccination in this cell population (Figure 12D).

Similar patterns were observed in the phenotype of the wider CD8 T-cell populations, although a trend toward greater frequencies of cytokine-producing TranEM cells in comparison to TCM cells was also observed (Figure 12E–G). Comparison of CD8 memory populations between the vaccination groups revealed that significantly greater frequencies of antigen-specific TranEM (*p* = 0.05) and TCM (*p* = 0.03) populations were measured in PBMCs isolated from aerosol BCG-vaccinated animals 20 weeks following vaccination, indicating that aerosol BCG induced antigen-specific CD8 T-cell populations that remained in circulation within a week of the *M. tuberculosis* challenge delivered at study week 21 (Figure 12E,F).

## 4. Discussion

TB remains a leading cause of global mortality and is responsible for more deaths than any other infectious disease [38]. The continued emergence of multi-drug resistant strains of *M. tuberculosis,* and the overlap between the HIV and TB epidemics, necessitates a more efficacious vaccination regimen for the control of TB. In the absence of a validated correlate of protection against TB, highly characterised animal models that can be used to differentiate promising vaccine candidates and refinements to the current vaccination regimen are required. We have previously described the development of an ultra-low dose aerosol challenge model in rhesus and cynomolgus macaques using an inoculum of the *M. tuberculosis* Erdman strain that is representative of natural infection [6]. The aerosol *M. tuberculosis* challenge dose applied in this study was comparable to these prior experiments, with a median presented dose quantified as 23 CFU, which equates to an estimated 3 CFU retained within the lung [21], demonstrating the reproducibility of the ULD aerosol challenge system we have developed. The selection of an appropriate *M. tuberculosis* challenge strain and dose is critical to pre-clinical TB vaccine evaluation strategies. Our goal in developing the ULD aerosol *M. tuberculosis* challenge model was to establish a system that can reproducibly deliver a challenge inoculum that is sufficient to cause disease in all animals, but not so overwhelming that a promising vaccine may be rejected because it is unable to provide immunity against a level of disease that is not typical of natural infection. In this study, we report the application of this model for the assessment of TB vaccination regimens and demonstrate the capacity to measure vaccination mediated differences in disease pathology and *M. tuberculosis* bacterial load afforded by BCG vaccination applied by aerosol or intradermal injection. Furthermore, variation in the pattern of extra-thoracic disease was measured between the groups that received BCG vaccination delivered parenterally or to the mucosal surfaces of the lung as an aerosol, demonstrating the sensitivity of the model to detect subtle differences in disease parameters and differentiate between similar vaccination strategies.

In comparison to higher dose *M. tuberculosis* challenge systems [3,24,39,40], the disease that develops in rhesus macaques following ULD aerosol *M. tuberculosis* exposure follows a slower pattern of progression [6]. This feature offers distinct advantages for evaluation of vaccine efficacy, including the reduced occurrence or delayed onset of progressive disease that can lead to a requirement for animals to be euthanised on welfare grounds. As well a notable refinement in terms of animal welfare, harmonisation of the timespan available for the development of infection and features of tuberculous disease leads to improved comparability of vaccine efficacy readouts between treated and untreated control groups and is consequently a valuable improvement to in vivo efficacy study design. These key features of the rhesus macaque ultra-low dose aerosol challenge model combine to provide a reproducible and sensitive system for assessment of the protective efficacy of novel TB vaccine candidates and regimens.

We have recently described the mucosal and systemic cellular immune response induced by BCG delivered as an aerosol using a portable VMN [19], and, in this study, we set out to directly compare the protective efficacy against ULD *M. tuberculosis* challenge imparted by aerosol and ID BCG vaccination. Consequently, mucosal immune responses were not interrogated to avoid potential interference with lung immunity prior to the aerosol *M. tuberculosis* challenge, and comparative immunology was focused on systemic response profiles.

It was evident from analysis of the frequency of antigen-specific cytokine-producing cells measured by ELISpot and flow cytometry, as well as the secretion of cytokine and chemokine markers quantified by multiplex bead array, that the onset of the systemic adaptive cellular immune response was significantly delayed following aerosol BCG vaccination, although, the peak and duration of these responses were comparable between the vaccination groups. For example, IFN-γ SFU frequencies were statistically equivalent immediately prior to aerosol challenge with *M. tuberculosis,* indicating that the delayed onset of vaccine-induced systemic immunity is unlikely to have interfered with a subsequent anamnestic response upon exposure to *M. tuberculosis*. Indeed, the frequency of mycobacteria-specific IFN-γ secreting cells increased concurrently in the aerosol- and ID-vaccinated groups following *M. tuberculosis* infection, albeit at a significantly slower rate than was seen in unvaccinated animals, which is likely to be indicative of the improved control of infection imparted by BCG vaccination delivered by either route [3,24]. Similarly, the functional profile of CD4 and CD8 T-cell subsets induced by aerosol or ID BCG vaccination were comparable when measured by multiparameter ICS assay, where a trend for delayed detection of antigen-specific cytokine-producing T-cell populations in aerosol BCG vaccinated animals was also apparent. However, interrogation of the memory phenotype of antigen-specific T-cell populations revealed that whilst cytokine-producing CD4 and CD8 populations were detected across the memory T-cell axis (encompassing TCM–TEM phenotypes) in both vaccination groups, TCM and TransEM CD8 T-cell populations induced by aerosol BCG vaccination were significantly higher than in ID BCG-vaccinated animals twenty weeks after vaccination. The continued presence of circulating antigen-specific TCM and TransEM CD8 T-cells within a week of aerosol *M. tuberculosis* challenge suggests that these cells may have contributed to the disease control observed in aerosol BCG-vaccinated macaques.

The variable efficacy of BCG vaccination, when delivered as an intradermal injection, is well documented in humans [8] and in preclinical models, including primates [24,41,42]. In common with previous reports, our experiment corroborates that there is variability in the degree of protection afforded to rhesus macaques by ID BCG vaccination, although a comparison between groups revealed that levels of disease pathology were significantly reduced in both ID and aerosol BCG-vaccinated groups relative to unvaccinated animals, providing a valuable and relevant benchmark against which novel vaccination regimens can be assessed. Aerosol BCG vaccination delivered in the laboratory setting has been shown previously to be immunogenic and to afford enhanced protection against inhaled *M. tuberculosis* infection in the rhesus macaque [16,19]. The availability of portable vibrating mesh nebuliser (VMN) technology has made the proposition of a widespread aerosol BCG vaccination campaign a viable alternative to needle-based vaccine delivery. With this comparison of the immunogenicity and efficacy of VMN delivered aerosol- and parenterally-delivered BCG, we have shown that the total level of disease pathology that developed in ID and aerosol BCG-vaccinated animals was equivalent following ULD *M. tuberculosis* challenge, although pathology measured in extrapulmonary tissues was significantly reduced by aerosol-delivered BCG vaccination. Similarly, the level of viable *M. tuberculosis* quantified in extra-thoracic (secondary) sites of infection was significantly reduced by aerosol BCG vaccination relative to unvaccinated animals, whereas the reduction in bacterial load measured in ID BCG-vaccinated animals failed to reach significance. Although these levels of efficacy appear relatively modest in comparison to those reported from historical studies where shorter vaccination to challenge intervals and the less virulent H37Rv strain of *M. tuberculosis* challenge inoculum were employed [16], our findings provide verification that aerosolised BCG delivered by VMN can provide a significant level of protection to rhesus macaques against *M. tuberculosis* challenge which is at least equivalent, and by some measures (such as reduced extrapulmonary dissemination and disease) superior, to intradermally delivered vaccination. Similarly, we note that the levels of protection demonstrated herein are comparable to those recently reported by Darrah et al. who used an alternative VMN device, dose and preparation of BCG, and *M. tuberculosis* challenge system, although their experiments were not powered to demonstrate vaccine efficacy relative to unvaccinated animals [43]. Therefore, we believe that there is a collective body of evidence supporting the further investigation of aerosol delivered BCG vaccination as an alternative to the current needle-based vaccination policy, although further optimisation of vaccination dose and refinement of the aerosol delivery apparatus to ensure efficient deposition and targeting of the vaccine should be investigated [44]. We note with interest the recent phase I clinical trial applications centred on VMN-delivered aerosol BCG [45,46], which may produce the requisite safety data to take this approach forward into further clinical studies.

## Figures and Tables

**Table 1:** Aerosol challenge doses of *M. tuberculosis* delivered to rhesus macaques.

Group	Vaccine and Route	Animal Identification Number	Presented Dose (cfu)	Estimated Retained Dose (cfu)
Group A	BCG Aerosol	U12	23	3
		U26	22	3
		U41	23	3
		U46	25	4
		U58	23	3
		U66	25	4
Group B	BCG ID	U18	22	3
		U28	27	4
		U31	21	3
		U56	24	3
		U72	23	3
Group C	No vaccine	U15	19	3
		U22	18	3
		U36	23	3
		U49	23	3
		U57	26	4
		U60	23	3
		U73	21	3
Study median			23	3

**Table 2:** Occurrence of microscopic changes associated with *M. tuberculosis* infection.

Group	Vaccine and Route	Animal ID	Pulmonary			Extra-Pulmonary Tissues			Lymph Nodes	
			Lung Lesion Type (1–6)	Bronchus Associated Lymphoid Tissue Involvement	Multi-Nucleated Giant Cells	Spleen	Liver	Kidney	Lung Associated	Peripheral
				(*n* of 7 Lung Lobes)		Present (+) or Absent (-)				
Group A	BCG Aerosol	U12	1, 2, 4, 5	0	5	-	-	-	+	-
		U26	1, 2, 4, 5, 6	0	6	+	+	-	+	-
		U41	1, 2, 4, 5, 6	0	7	+	+	+	+	-
		U46	1, 4, 5, 6	1	7	-	-	-	+	-
		U58	1, 2, 4, 5	3	7	-	-	-	+	-
		U66	2, 3, 4, 5, 6	0	6	-	+	+	+	-
Group B	BCG ID	U18	1, 2, 4, 5, 6	1	5	-	+	+	+	+
		U28	1, 2, 3, 4, 5, 6	3	7	+	+	+	+	-
		U31	1, 2, 4, 5, 6	1	6	+	+	+	+	+
		U56	1, 4, 5, 6	0	3	+	+	-	+	+
		U72	1, 4, 5, 6	0	4	+	+	-	+	-
Group C	No vaccine	U15	1, 4, 5, 6	0	5	+	+	+	+	+
		U22	1, 2, 4, 5, 6	0	6	+	+	+	+	+
		U36	1, 2, 4, 5, 6	1	6	+	+	+	+	-
		U49	1, 2, 3, 4, 5, 6	0	7	+	+	+	+	-
		U57	4, 5, 6	0	4	-	-	+	+	+
		U60	1, 2, 4, 5, 6	0	5	+	+	+	+	+
		U73	1, 4, 5, 6	0	7	+	+	+	+	+

## Supplementary Materials

The following are available online at https://www.mdpi.com/1999-4923/12/5/394/s1, Figure S1: PPD-specific production of individual cytokines measured by intra-cellular cytokine staining. Plots A, B, C and D show the frequency of IFN-γ, IL-2, TNF-α and IL-17 measured in CD4 T-cells; plots E, F, G and H show cytokine production measured in CD8 T-cells. Box plots display the group median frequency of cytokine-producing cells +/- interquartile range with minimum and maximum values shown by whiskers. Dots represent the frequency of cytokine-producing cells measured in individual animals. BCG vaccination is indicated by a dotted line at study week zero.
