# Supplementary material for: Protective Efficacy of Inhaled BCG Vaccination Against Ultra-Low Dose Aerosol M. tuberculosis Challenge in Rhesus Macaques
Source: Pharmaceutics. 2020 Apr 25;12(5):394. doi: 10.3390/pharmaceutics12050394 (PMC7284565; doi:10.3390/pharmaceutics12050394)
Supplement: Supplementary file 1 [file pharmaceutics-12-00394-s001.pdf]

pharmaceutics

MDPI

Supplementary Materials: Protective Efficacy of Inhaled BCG Vaccination Against Ultra-Low Dose Aerosol *M. tuberculosis* Challenge in Rhesus Macaques

Andrew D. White, Charlotte Sarfas, Laura S. Sibley, Jennie Gullick, Simon Clark, Emma Rayner, Fergus Gleeson, Martí Català, Isabel Nogueira, Pere-Joan Cardona, Cristina Vilaplana, Mike J. Dennis, Ann Williams and Sally A. Sharpe

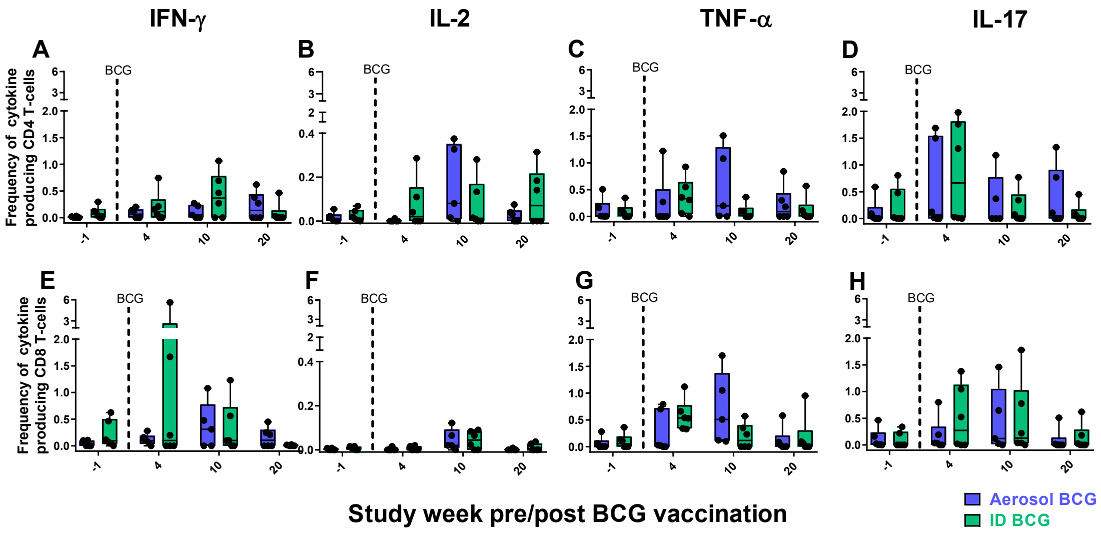

Figure S1. PPD-specific production of individual cytokines measured by intra-cellular cytokine staining. Plots A, B, C and D show the frequency of IFN-γ, IL-2, TNF-α and IL-17 measured in CD4 T-cells; plots E, F, G and H show cytokine production measured in CD8 T-cells. Box plots display the group median frequency of cytokine-producing cells +/- interquartile range with minimum and maximum values shown by whiskers. Dots represent the frequency of cytokine-producing cells measured in individual animals. BCG vaccination is indicated by a dotted line at study week zero.
